# Amine-Functionalized Clays as Solid Sorbents: High-Pressure CO_2_ Sorption Testing and Characterization

**DOI:** 10.1021/acsomega.5c06923

**Published:** 2026-01-13

**Authors:** Jennifer Narváez, Ernesto Bastardo-González, Edward E. Ávila, Alex Palma-Cando, Pamela Galarraga, Víctor H. Guerrero, Marvin Ricaurte

**Affiliations:** † Grupo de Investigación Aplicada en Materiales y Procesos (GIAMP), School of Chemical Sciences and Engineering, Yachay Tech University, Hacienda San José s/n y Proyecto Yachay, Urcuquí 100119, Ecuador; ‡ Research Development and Innovation Department, UNACEM Ecuador, Sector Perugachi Km 71 1/2 vía a Selva Alegre, Otavalo 110100, Ecuador; § Department of Materials, Escuela Politécnica Nacional, Ladrón de Guevara E11-253, Quito 170525, Ecuador

## Abstract

In this work, the CO_2_ sorption capacity of both raw and amine-functionalized clays was investigated. For this purpose, samples of clay-based materials intended for industrial and artisanal applications were collected in the province of Imbabura, Ecuador. These raw materials were functionalized with monoethanolamine (MEA) and ethylenediamine (EDA) mixtures to enhance the affinity for carbon dioxide. CO_2_ sorption testing was conducted in a high pressure, nonstirred system with an initial pressure of 3550 kPa and room temperature (25 °C). CO_2_ sorption capacity was quantified by monitoring the pressure drop resulting from CO_2_ uptake. Comprehensive characterization of the clay-based materials was carried out using nitrogen physisorption analysis, X-ray diffraction (XRD), Fourier transform infrared spectroscopy (FTIR), thermogravimetric analysis (TGA), and X-ray fluorescence (XRF) to determine the textural properties, crystalline phases, physicochemical properties, and functional groups present in the samples. The results demonstrated that amine-functionalized clays exhibited higher CO_2_ sorption capacity, with improvements of up to 442.85% compared to raw materials. Notably, the amine-functionalized clays intended for artisanal usage achieved a maximum capacity of 3.125 mmol CO_2_/g. These findings confirmed that amine functionalization favors CO_2_ capture, offering a viable and scalable alternative to mitigate the impacts of the rising atmospheric CO_2_ emissions.

## Graphical abstract

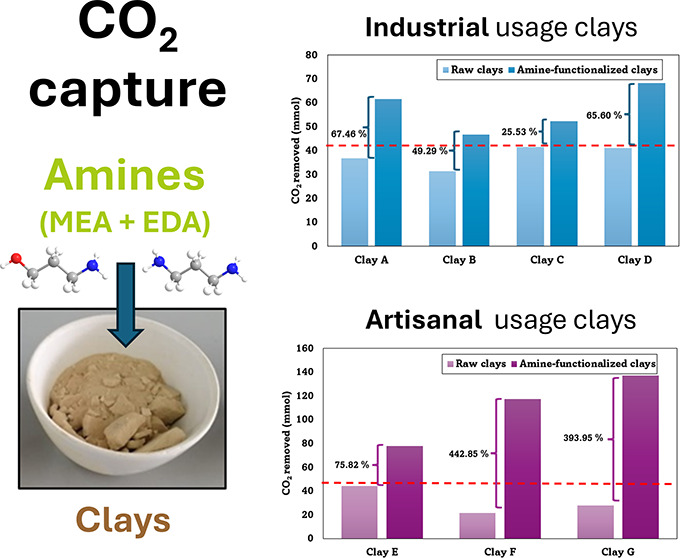

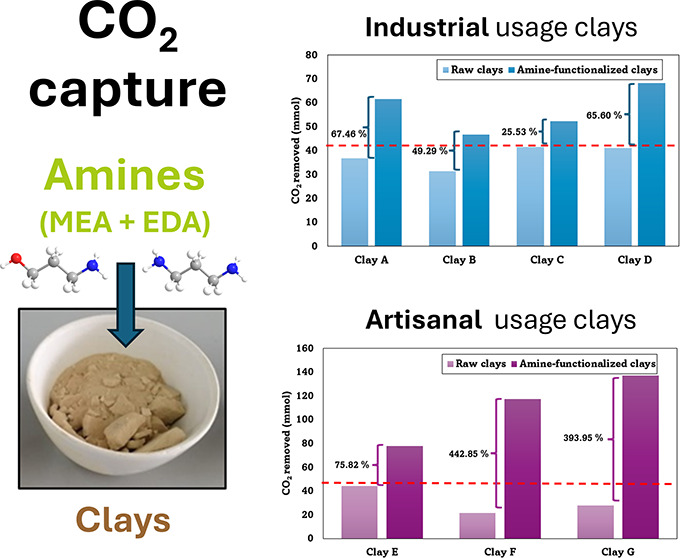

## 1 Introduction

The increase in atmospheric carbon dioxide (CO_2_) concentrations caused by human activity is the main factor responsible for the intensification of the greenhouse effect and the resulting climate change.^1–3^ This is due primarily to the combustion of fossil fuel for energy generation, deforestation, and agricultural activities.^4,5^ According to reports from the Intergovernmental Panel on Climate Change (IPCC), human activities—mainly burning fossil fuel and industrial processes—have raised atmospheric CO_2_ concentrations from approximately 280 ppm (280 ppm) in the preindustrial era to more than 410 ppm today.^6^ This increase has led to phenomena such as rising global temperatures, changing weather patterns, and an increase in the frequency of extreme weather events.^7–9^ Developing effective technologies to mitigate CO_2_ emissions has become a scientific and technological priority.^10–17^

CO_2_ capture though the use of solid sorbents is a promising technology that has attracted intense attention from academia and industry in recent decades.^17–21^ Solid sorbents have become a compelling alternative to traditional liquid-based capture processes such as aqueous amine solutions.^22–29^ These solid sorbents can be porous materials, such as zeolites and clays, which offer advantages such as lower environmental impact, ease of regeneration, and the ability to operate under more varied process conditions, including high pressures and elevated temperatures.^22,30–34^ The distinctive crystalline structure of clays provide these materials unique properties such as high cation exchange capacity, swelling behavior, specific surface area, porosity, availability of active sites, and good CO_2_ sorption capacity.^35–37^ An essential structural characteristic of clays is that they can contain different cations (e.g., Mg^2+^, Na^+^, Ca^2+^) in the interlayer space. In particular, functionalized clays exhibit great potential due to their good scalability and their ability to be chemically modified.^38–40^ The functionalization of clays with amines has been used as the primary method to improve the CO_2_ sorption capacity.^41–43^ This process can generally be carried out thought physical methods (such as impregnation) or chemical methods (such as grafting).^44,45^ Amines are the most used modifiers due to their high reactivity with CO_2_.^46–49^ Monoethanolamine (MEA) and ethylenediamine (EDA) react with CO_2_ through an acid–base mechanism, forming stable bonds under specific pressure and temperature conditions.^42,50^

Clay materials are categorized by their industrial and artisanal uses. For industrial purposes, clays are typically used in the ceramic industry to produce porcelain, fine and coarse ceramics, terracotta, electroceramics, tiles, and refractories.^51,52^ Clays have also been used as supplementary cementitious material in Portland cement concretes.^53^ Conversely, clays intended for artisanal purposes are typically utilized to create ceramics, decorative figures, and kitchen utensils.^54^ However, the fundamental behavior of both types of clay follows the same principles, given that they possess properties like high cation exchange capacity, binding capacity, plasticity, a strong tendency to react with organic compounds, and thixotropy.^55–58^ Due to the presence of manganese, iron, and potassium in their structure, the color, purity, and efficiency in acidic and/or basic conditions, make them optimal for use in the sorption of CO_2_.^49,53^

Recently, Altaf et al.^59^ explored the potential of clay minerals as sorbents for CO_2_ due to their abundance and low cost. High specific surface area and porosity give the clays remarkable CO_2_ sorption capacity since these characteristics provide abundant active sites for interaction with CO_2_ molecules.^45^ Impregnation with amines and other chemical treatments increase their capacity and stability for carbon capture. When raw clays are used without any treatment, such as smectites and kaolinite, they show a low CO_2_ sorption capacity (0.11–0.14 mmol CO_2_/g) at room temperature and atmospheric pressure.^60^ On the other hand, clays such as bentonite, montmorillonite, and saponite show CO_2_ sorption capacity between 0.23 and 0.34 mmol CO_2_/g at 45 °C.^42^ Smectite-like clays can sorb CO_2_ molecules in their interlayer region as a function of the degree of hydration.^61^ Another clay group that demonstrated a significant CO_2_ sorption capacity includes the fibrous phyllosilicates, such as sepiolite and palygorskite at ratios of 1.45 mmol CO_2_/g and 0.41 mmol CO_2_/g at 25 °C and atmospheric pressure, respectively.^62^ This group can act as a molecular sieve, a function that is shared with zeolites. Considering these examples, it can be established that temperature plays a vital role in the CO_2_ sorption capacity of clay-based materials.^59,63^

Many of the clay-based materials found in Ecuador can be used as solid sorbents,^64^ with some of them recognized as excellent alternatives for the sorption of CO_2_.^65^ Herein, a group of Ecuadorian clays were selected and amine-functionalized to increase their capacity to sorb this acidic gas. The sorbent samples were subjected to CO_2_ sorption tests, carried out under isochoric conditions, including an initial pressure of 3550 kPa and room temperature (25 °C). In addition, the clay-based materials were characterized using nitrogen physisorption analysis, X-ray diffraction (XRD), infrared spectroscopy (FTIR), thermogravimetric analysis (TGA), and X-ray fluorescence (XRF). This allowed accurate assessment the amount of CO_2_ retained in the material and the determination of the textural properties, crystalline phases, physical-chemical properties, and functional groups present in the samples.

## 2 Methods

### 2.1 Samples of Clay-Based Materials

The clay-based materials selected for the CO_2_ sorption tests are from the province of Imbabura, Ecuador (Figure S1). The samples were classified according to their recurrent use: industrial and artisanal. The industrial usage samples were provided by the company UNACEM, which utilizes clays as raw materials or additives in cement manufacturing.^66^ Artisanal clays are commonly used to manufacture kitchen utensils and household crafts.^67,68^ Depending on the current utility, the samples of clay-based materials were divided into two groups: four (4) samples for industrial usage (labeled A, B, C, and D) and three (3) for artisanal usage (labeled E, F, and G).

The two groups of clay-based materials showed slight differences in their physical properties and visually apparent characteristics, which were the primary basis for assigning the treatments they underwent before the CO_2_ sorption tests. The artisanal clays exhibited greater plasticity in their original state compared to the industrial clays. This plasticity can be attributed to the high natural content of minerals combined with impurities such as organic matter.^69^ This characteristic should facilitate a more uniform dispersion and mixing with functionalizing agents, such as amines, thereby improving their performance in CO_2_ sorption tests. In contrast, the original industrial clays presented a high content of sandy material in their raw state which is essential to consider. In this case, subjecting the clays to a washing process allows us to achieve sedimentation and stratification of the sandy material to recover most of the ultrafine particles. With this process, it is possible to eliminate impurities and increase cohesion and malleability.

### 2.2 Treatment and Amine-Functionalization of Clays: Protocols

The general scheme of the treatment and amine-functionalization of clay-based materials is shown in Figure 1. All clay-based samples (∼2000 g) were dried in an oven at 80 °C for 24 h. The sieving was carried out with a mesh to separate particles smaller than 1 mm. Only the fine material fraction was manually washed with tap water in appropriate containers. Here, the industrial usage clay samples were gently stirred to facilitate a good separation of the sandy particles from the clay ones. Once this process was completed, the material was decanted into large containers to ensure good precipitation of the particles. This precipitation took place over 12 h. Then, the precipitated material was sun-dried for 10 h. As a result, a differentiation of layers was clearly visible: a top layer (mainly clay), a middle layer (mixture of clay and sand), and a bottom layer (mainly sand). These different fractions were carefully separated with a spatula and dried in an oven at 80 °C for 24 h to eliminate any remaining moisture. The fractions of recovered layers were stored in packages. Then, each layer obtained from the industrial clays as well as the raw clays were tested to quantify the initial CO_2_ sorption capacity. These samples were previously calcined at 450 °C for 8 h to activate the material via the oxidation and decomposition of organic matter, the release of structural water, and the transition of clay compounds.^70^

**Figure 1:**
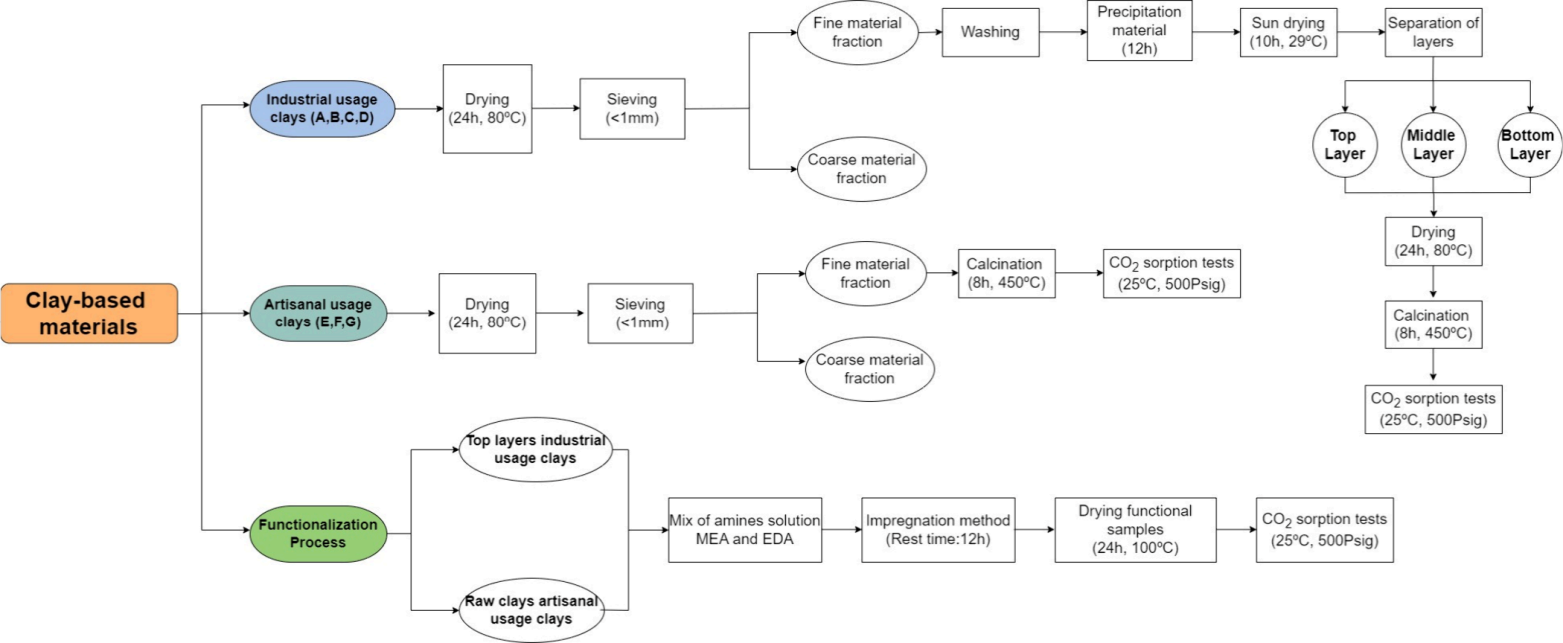
Treatment and amine-functionalization of clays: method outlook.

In addition, the top layer of the washed industrial clays as well as the artisanal clays were functionalized with amines using the wet impregnation method to increase the CO_2_ sorption capacity. A mixture of MEA and EDA (50 wt % each) solutions, was used for this purpose. The amine solution was deposited on the surface of each clay sample until it was completely wet. Then, the samples were left to rest at room temperature for 12 h to achieve a good impregnation of the amines in the clays. These clays were then subjected to oven drying at 100 °C for 24 h. Finally, the amine-functionalized samples were pulverized and subjected to CO_2_ sorption tests.

### 2.3 CO_2_ Sorption Testing Protocol

The CO_2_ sorption tests were performed using high-pressure reactors (Parr HPS Series 4790 pressure vessel systems) that consisted of two main components: a mobile head and a cylinder, with a nominal capacity of 100 mL. The reactor head included standard accessories, such as a pressure gauge and a thermocouple, allowing internal pressure and temperature monitoring by SpecView 3.1 data acquisition and control software. The reactors are made of C-276 alloy and specifically designed for batch operations under extreme conditions (pressure: max. 6900 kPa; temperature: max. 225 °C).

For the experimental configuration, 30–35 g of clay-based materials were loaded into the reactor. The temperature was set at 25 °C, and the system was pressurized up to 3550 kPa. The experimental test was run for 20 h to record data and for the pressure to reach equilibrium. Additional details on the equipment and experimental procedure for high-pressure CO_2_ capture testing can be found in recent studies by our research group.^71,72^

#### 2.3.1 Data Processing

The primary data, pressure (*P*) and temperature (*T*), was acquired based on time (*t*). In addition, using the Peng–Robinson equation of state (PR EoS),^73^ the compressibility factor (*Z*) was determined to calculate the moles of CO_2_ in the gas phase at any time (*n*_*t*_), using Equation 1.

$${PV}_{gas}=n_{t}ZRT$$ 1

The volume of gas (*V*_*gas*_) is obtained by subtracting the volume of the reactor (*V*_*r*_ = 100 *mL*) from the volume occupied by the mass of the clay-based materials (*V*_*clay*_), using Equation 2. *V*_*clay*_ was calculated from the density of each clay-based material (*ρ*_*clay*_). *ρ*_*clay*_ was estimated indirectly by measuring the mass (*m*_*clay*_) and volume of the samples. The density values are shown in Table S1.

$$V_{gas}=V_{r}-V_{clay}=V_{r}-\frac{m_{clay}}{\rho_{clay}}$$ 2

The CO_2_ captured (*n*_*CO*_2_ *removed*_) represents the portion of CO_2_ that goes from the gas phase to the clay-based materials. It was determined based on the difference between the amount of CO_2_ initially present in the gas phase (*n*_0_) and the amount of CO_2_ that remained over time (*n*_*t*_), by Equation 3.

$$n_{{CO}_{2}removed}=n_{0}-n_{t}$$ 3

The total quantity of CO_2_ removed, divided by the clay mass, gives the CO_2_ loading capacity per gram of clay-based material (*CO*_2_ *loading*) based on Equation 4:

$${CO}_{2}\;loading=\frac{n_{{CO}_{2}removed}}{m_{clay}}$$ 4

This made it possible to compare the CO_2_ sorption capacities between all the clay-based materials tested in this work with those reported in literature, stated as *mmol CO*_2_/*g clay*.

### 2.4 Physical and Chemical Characterization Methods

The characterization methods described below were used for samples under three conditions: unreacted samples (original clays), CO_2_-reacted samples without functionalization, and CO_2_-reacted samples with functionalization. This applied to both industrial and artisanal usage clays.

**Nitrogen Physisorption Analysis:** Nitrogen sorption tests were conducted to determine the specific surface area and pore volume of the clay-based materials. These analyses were conducted using a Quantachrome NOVAtouch 1LX instrument. For this purpose, the samples were vacuum degassed by heating at 80 °C at a rate of 10 °C/min and held for 24 h. Measurements were performed at 77 K, and at least 48 data points were used to plot the adsorption–desorption curves. Specific surface area was calculated using the Brunauer–Emmett–Teller (BET) method, while pore size was evaluated using the Barret-Joyner-Halenda (BJH) method.
**X-ray Diffraction (XRD):** A diffractometer for polycrystalline samples (Miniflex600 from Rigaku) with a D/teX Ultra detector and SmartLab Studio II software was used in this work. An X-ray generator operated at 40 kV and 15 mA, CuKα_1,2_ nonmonochromatized radiation source (sealed tube), 2θ scanning axis, 0.01° step, 5–100° 2θ scanning range, 10°/min speed, and D/tex Ultra2 detector in 1D scanning mode were used for data acquisition. Finally, the QUALX program was used for data processing, taking POW CODINO as the database.
**Fourier Transform Infrared Spectroscopy (FTIR):** The FTIR spectrometer was a Spectrum Two from PerkinElmer that has attenuated total reflectance (ATR) and a standard MIR LiTaO_3_ detector with SNR of 9,300:1. Its optical system features KBr windows operating over a spectral range of 8,300–350 cm^–1^ given an optimal resolution of 0.5 cm^–1^. The instrument is controlled using Spectrum 10TM software, which is also used to process the spectra collected. OriginLab software was used for data graph analysis.
**Thermogravimetric analysis (TGA)**: The equipment used was a TGA 55 model from TA Instruments, equipped with a 100 μL platinum crucible. The analysis was performed under oxidizing atmosphere conditions at a speed of 20 °C per minute, from 20 to 900 °C.
**X-ray Fluorescence (XRF)**: The XRF spectrometer used was an S8 Tiger from Bruker. It has a detection range covering elements from Na (Z = 11) to U (Z = 92) and uses an excitation source based on an X-ray tube with an Rh, Ag/Mo anode, Si Drift Detector (SDD), or proportional detectors to guarantee high resolution. In addition, it incorporated advanced software for automatic qualitative and quantitative analysis.

## 3 Results and Discussion

### 3.1 Treatment and Functionalization of Clays

Three well-differentiated layers were identified during the stratification process of industrial usage clays. The top layer presented a predominantly clay-based material with a light brown to beige color, a smooth texture, and very fine granules, almost imperceptible to touch. This material had high plasticity and retained a greater amount of water. The middle layer was a heterogeneous mixture of clay and sand with a darker brown tone. The grains in this layer were medium-sized, with a somewhat rough texture, and showed a gradual transition between the clay fineness and the sand granularity. Finally, the bottom layer was predominantly sand-based material, with larger particles and a rough texture. Its color varied between dark brown and light gray, depending on the proportion of minerals present. This layer was less plastic and denser. The segregation of these layers can be observed in Figure 2 for clay A. Table 1 reflects the results of the fractions recovered from each layer of the 4 industrial usage clays.

**Figure 2:**
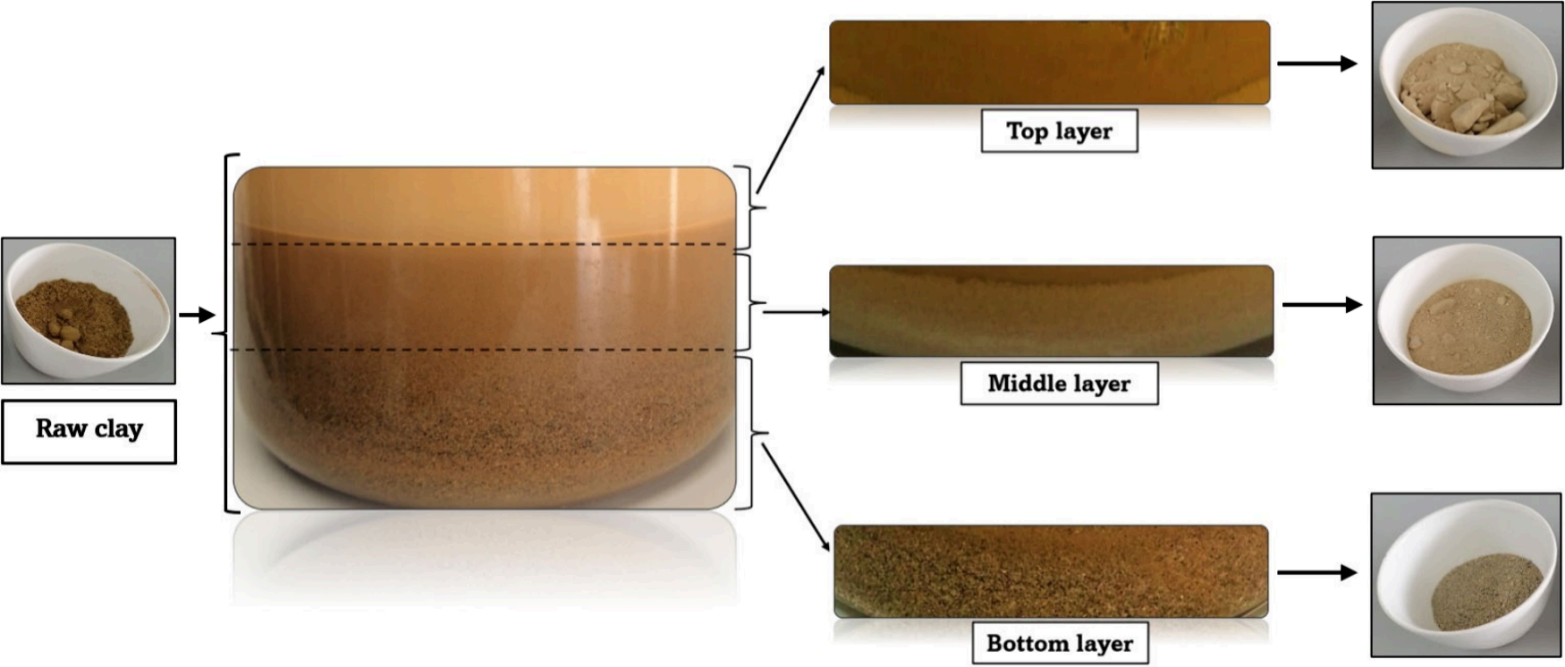
Stratification of layers in industrial usage clays after washing and sun-drying.

**Table 1:** Fractions Recovered from Each of the Industrial Usage Clay Layers after Stratification

Samples	Top layer (wt %)	Middle layer (wt %)	Bottom layer (wt %)
Clay A	16.09	9.76	71.98
Clay B	25.38	25.18	48.69
Clay C	18.37	25.75	54.37
Clay D	9.58	-^a^	87.44

a (−) Not present.

The plasticity of clay-based materials is affected by their composition (types of clay minerals, proportion of nonplastic minerals, etc.), organic substances, specific surface area, state of dispersion of particles, particle size distribution, and water characteristics (viscosity and surface tension).^35,74,75^ A clay–water system with high plasticity requires greater force for deformation and deforms more extensively with cracking, while a system with low plasticity deforms easily and is more prone to breaking. As the water content in clay minerals increases, plasticity also increases, depending on the nature of the clay.^76^ When water is added to dry clay, the cohesion increases and tends to the maximum after displacing the air from the pores between the clay particles. When water reaches the pores, it causes the formation of a body with high resistance to the elastic limit, resulting in cracking or breaking due to deformation.^77^ Figure S2 illustrates the plasticity of different layers of industrial usage clays. The top layer showed the highest plasticity, which could indicate a high concentration of clay-based materials in this layer. In the case of the artisanal usage clays, there were no layer divisions. Therefore, raw samples were considered in this case with no further purification.

#### 3.1.1 Amine-Functionalization Process: Wet Impregnation Method

MEA and EDA amines, 50 wt % each, were applied in the functionalization process (Figure S3). The amines have a high affinity for carbon dioxide.^78,79^ Both contain amino groups (−NH_2_) that react with CO_2_ to form carbamates or bicarbonates, thus improving the sorption capacity.^80^ When the amines are used to functionalize clays, these amines can easily bind to the surface due to electrostatic interactions, hydrogen bonds, or ion exchange.^81,82^ Being low molecular weight amines, they facilitate their dispersion in the pores or surface of clay minerals, increasing the number of active sites for CO_2_ capture.^83^ Table S2 presents data on the mass increase of the clays from amine impregnation, with an average of 5.57%. Some studies suggest that amino groups in amine-modified clays play a significant role for improved CO_2_ sorption.^59,84–87^

### 3.2 CO_2_ Sorption Tests

#### 3.2.1 Industrial Usage Clays

At 25 °C, the CO_2_ sorption capacities of raw clays, the individual stratified layers, and the amine-functionalized top layers were analyzed. Figure 3a shows the partial pressure and the captured CO_2_, as a function of the sorption time for the raw clay and the different layers of clay A. It indicates that the top layer showed a greater pressure drop over time and had a higher amount of CO_2_ removed than the other layers until reaching values of ΔP: 470 kPa and 25.513 mmol of CO_2_ removed at the end of the experiments. This behavior suggested that the top layer of clay A interacts more with CO_2_ due to the diffusion of this acidic gas in the layers.^88,89^ Similar behaviors were observed in the other samples, B, C, and D. Considering that the top layers showed higher CO_2_ sorption capacities, the amine-functionalization process and characterization was performed only for the top layers.

**Figure 3:**
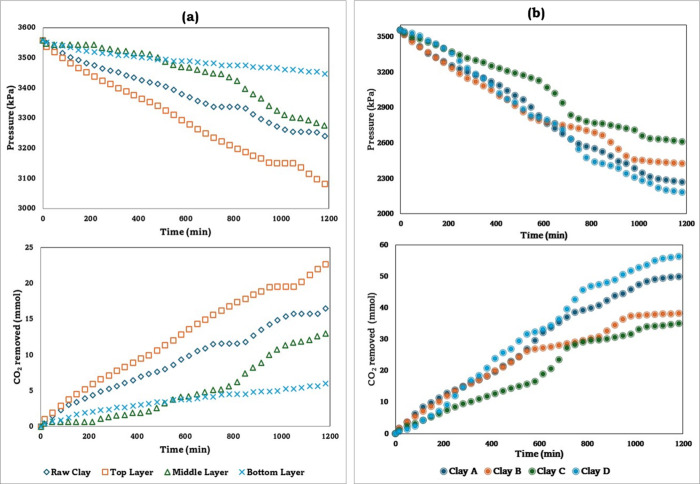
CO_2_ sorption for the industrial usage clays—pressure drop and CO_2_ removed. (a) Clay A: raw clay and stratified layers (nonfunctionalized samples). (b) Amine-functionalized clays (top layers).

Figure 3b shows the partial pressure and CO_2_ captured as a function of time for the top layers of the amine-functionalized clays. Once functionalized, the industrial usage clays almost doubled their CO_2_ sorption capacity compared to the nonfunctionalized ones. This phenomenon not only suggested the successful impregnation of the amine molecules on the clays structure (see Figure S3 and Table S2) but also the availability of the amino groups to capture CO_2_. Clay samples A and D showed better results compared to samples B and C, presumably due to a higher CO_2_ affinity. However, all the top layer samples for the industrial usage clays showed good results once amine-functionalized, and this may be due to the chemical composition of the material, cation exchange capacity, and chemical affinity toward CO_2_.^45,90^ The quantitative results obtained for the CO_2_ capture from these samples are reflected in Table 2.

**Table 2:** CO_2_ Capture Data for Industrial Usage Clays

Samples		Final pressure (kPa)	CO_2_ removed (mmol)	CO_2_ loading (mmol CO_2_/g)
Clay A	Raw clay	3191.58	18.846	0.623
	Top layer	3019.21	25.513	0.836
	Middle layer	3274.32	13.001	0.431
	Bottom layer	3446.68	5.994	0.198
	Amine-functionalized clay^a^	2253.89	50.346	1.396
Clay B	Raw clay	3281.21	12.510	0.411
	Top layer	2950.26	21.511	0.710
	Middle layer	3184.68	15.239	0.499
	Bottom layer	3391.53	7.591	0.247
	Amine-functionalized clay^a^	2412.47	38.624	1.049
Clay C	Raw clay	3150.21	22.375	0.733
	Top layer	2766.52	30.122	0.980
	Middle layer	3288.10	12.472	0.415
	Bottom layer	3124.70	21.887	0.720
	Amine-functionalized clay^a^	2371.10	42.767	1.186
Clay D	Raw clay	3412.21	8.084	0.267
	Top layer	2860.63	28.194	0.933
	Bottom layer	3276.38	15.540	0.571
	Amine-functionalized clay^a^	2178.05	56.465	1.547

a The top layer of each clay was amine-functionalized to enhance the CO_2_ sorption capacity.

#### 3.2.2 Artisanal Usage Clays

In Figure 4a, the partial pressure and the captured CO_2_ are represented as a function of the sorption time. The behavior of these materials can similarly be attributed to the same reasons presented in the industrial usage clays. Greater CO_2_ diffusion in the laminar structure of the clays contributes to the retention of CO_2_ molecules. For the raw clay samples F and G, there may be fewer pores or blockage, which prevents good gas sorption. Therefore, the diffusion process is slower, and early saturation occurs, reducing CO_2_ capture. For these samples, the amine-functionalization was conducted for each raw sample, as observed in Figure 4b. Once the samples are functionalized, the CO_2_ sorption capacity increases almost three times compared to the raw clay samples. This can be attributed to the same causes and characteristics described for the industrial-use samples. Interestingly, there is a proportional relationship between the percentage impregnation of amines and the CO_2_ loading in the functionalized artisanal clays showing the highest values for clay G. The quantitative results obtained for the CO_2_ capture from these samples are reflected in Table 3.

**Figure 4:**
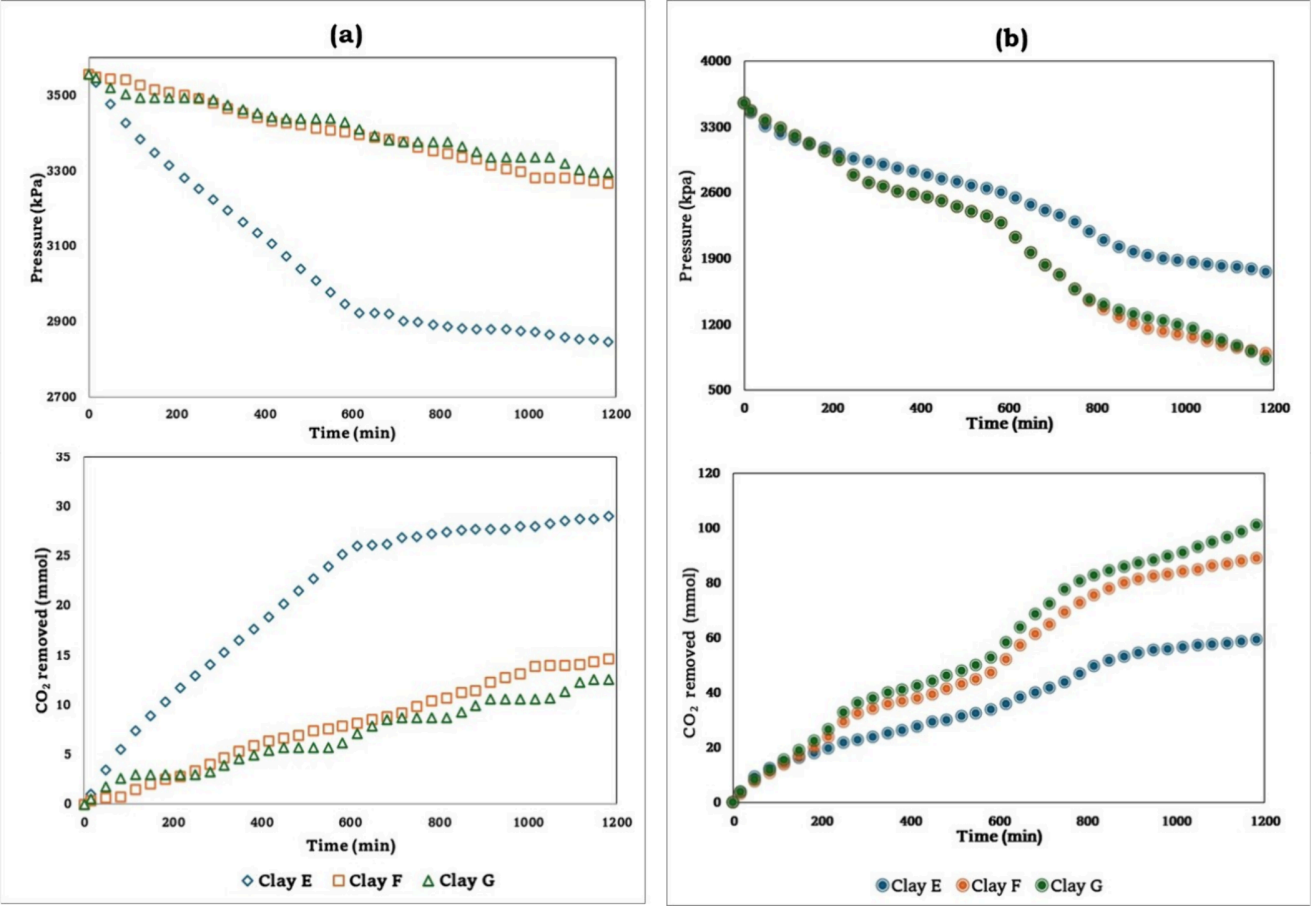
CO_2_ sorption for the artisanal usage clays—pressure drop and CO_2_ removed. (a) Raw clays (nonfunctionalized samples) and (b) amine-functionalized clays.

**Table 3:** CO_2_ Capture Data for Artisanal Usage Clays

Samples		Final pressure (kPa)	CO_2_ removed (mmol)	CO_2_ loading (mmol CO_2_/g)
Clay E	Raw clay	2805.47	30.600	1.008
	Amine-functionalized clay	1660.94	62.369	1.763
Clay F	Raw clay	3260.53	15.001	0.491
	Amine-functionalized clay	681.89	94.351	2.663
Clay G	Raw clay	3150.21	19.275	0.634
	Amine-functionalized clay	488.83	110.896	3.125

The CO_2_ loading results for the raw clays for artisanal usage clays reflected consistent and acceptable values reported for clay-based sorbents,^45,90^ as the results fell within a range of 0.491–1.008 mmol CO_2_/g. In contrast, once the samples were amine-functionalized, the CO_2_ sorption capacity increases significantly, reaching values between 1.763 and 3.125 mmol CO_2_/g, thus confirming the effectiveness of the functionalization proposed in this work. Jedli et al.^83^ reported CO_2_ sorption capacity values 1.36–1.59 mmol CO_2_/g for functionalized clays tested at atmospheric pressure.

#### 3.2.3 Industrial Usage and Artisanal Usage Clays: Comparison

Figure 5 shows the percentage improvement in the CO_2_ sorption capacity in the amine-functionalized clays compared to the raw samples.

**Figure 5:**
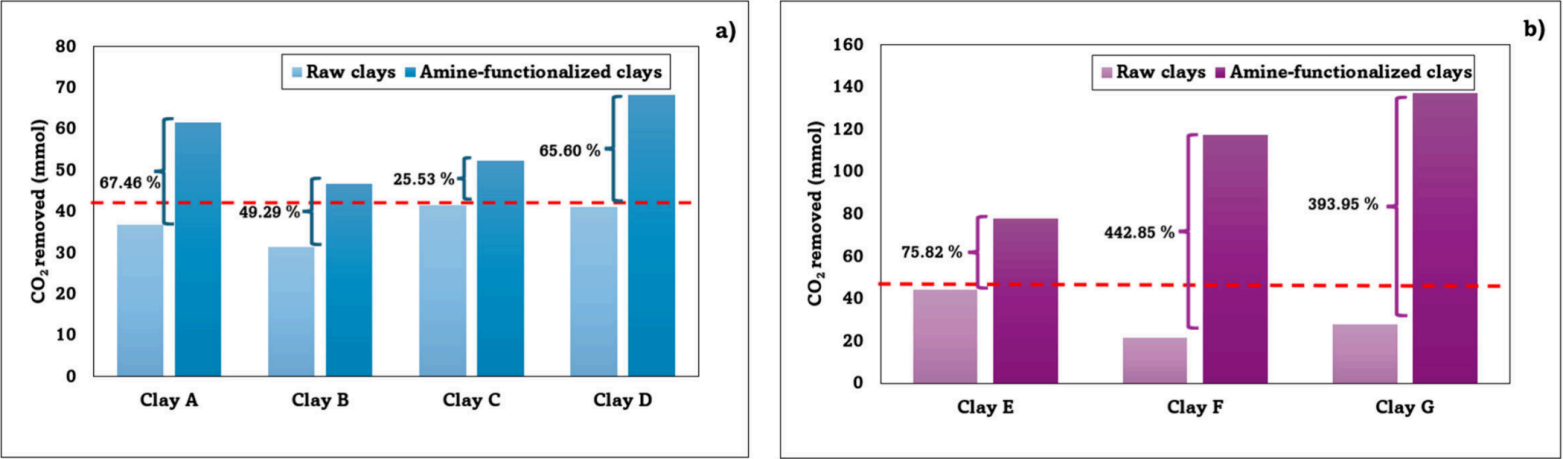
Comparison of percentage increase in the CO_2_ sorption capacity of raw clays and amine-functionalized clays: (a) industrial usage and (b) artisanal usage. (NOTE: For the industrial clays, the comparison was performed on the top layer, as it exhibited the highest CO_2_ adsorption capacity).

The amine-functionalization of the clays proved to be favorable in all the analyzed cases, achieving a significant increase in CO_2_ capture capacity. In industrial usage clays, although all showed improvements, clay A stood out with a 67.46% increase, which showed the highest percentage of improvement in this group. Similarly, in artisanal usage clays, all cases were positive, with notable increases in clays F and G, which reached 442.85% and 393.95% improvements in their CO_2_ sorption capacity, respectively. When contrasting the results between the two groups of these tested clay-based materials (industrial usage and artisanal usage), it was quite evident that the differences are in the functionalization efficiency and their absolute CO_2_ capture capacities. Industrial usage clays showed much lower percentage increases compared to the artisanal usage clays.

Figure S4 contrasts the CO_2_ loading with the mass increase resulting from the amine impregnation. In general terms, a direct correspondence was observed between the amount of impregnated amine in the clays and the amount of CO_2_ captured.

This, again, suggests that the amine impregnation is favorable for increasing CO_2_ capture, with this effect being more considerable in the artisanal amine-functionalized clay samples.

### 3.3 Characterization Results

Considering the performance observed in the CO_2_ sorption capacity testing, the physicochemical characterization of the industrial usage clays was focused exclusively on the top layer, whereas all the artisanal usage clays were characterized. As previously indicated, these samples were characterized under three distinct conditions: unreacted samples (original clays), CO_2_-reacted samples without functionalization, and CO_2_-reacted samples with functionalization.

#### 3.3.1 Nitrogen Physisorption Analysis

The specific surface area of porous materials is commonly evaluated by applying the BET model to gas sorption isotherms measured at different temperatures. Figure 6 shows nitrogen physisorption isotherms for unreacted samples. According to IUPAC’s classification,^91^ both the original and amine-functionalized clays exhibit type II isotherms, which are characteristic of clay-based materials.^92^ Type II isotherm is the normal form of isotherm obtained with a nonporous or macroporous adsorbent. The hysteresis observed at high relative pressures can be attributed to the stacking of the lamellar structures, which generates additional mesoporosity. This phenomenon was also reflected in the average pore radius for the clays with values between 3.78 and 25.20 nm (see Table 4), calculated using the BJH method.

**Figure 6:**
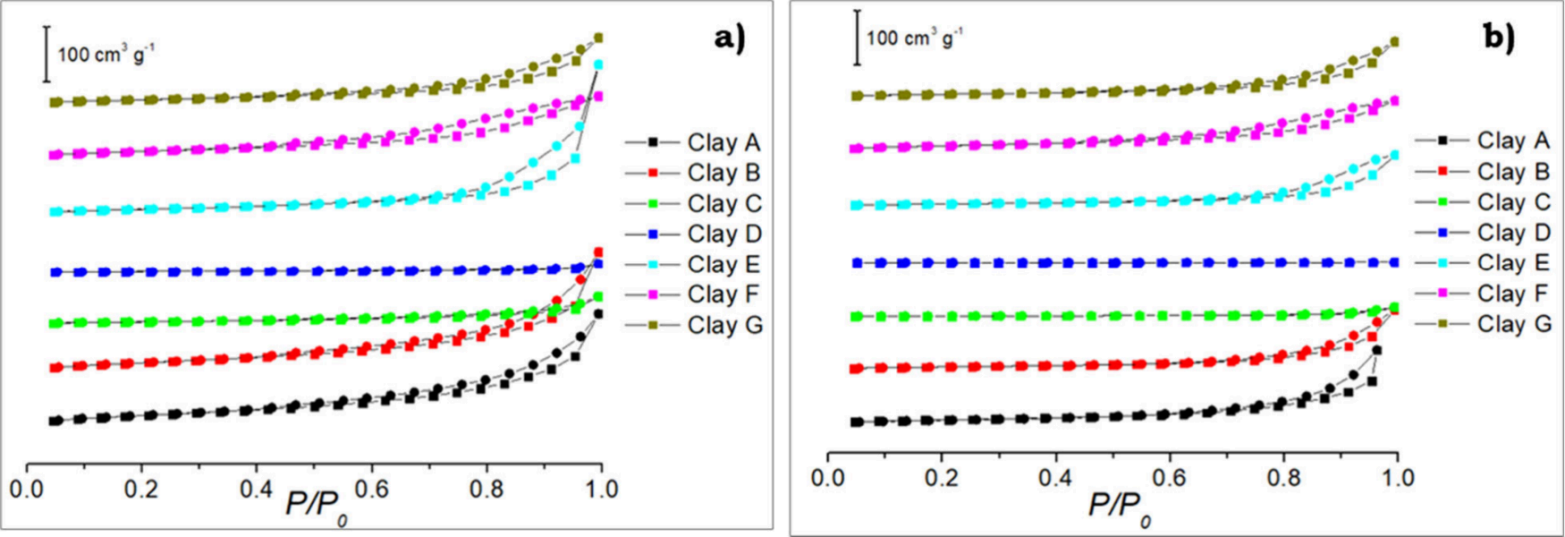
Nitrogen gas physisorption isotherms: (a) original clays and (b) amine-functionalized clays.

**Table 4:** Specific surface area and average pore radius of the raw and amino-functionalized clays

	Industrial clays				Artisanal clays		
	Clay A	Clay B	Clay C	Clay D	Clay E	Clay F	Clay G
Original clays (nonfunctionalized samples)							
Specific surface area (m^2^/g)	137	125	35.0	7.5	62	82	60
Average pore radius (nm)	5.08	5.92	5.04	7.23	14.13	4.69	6.74
Amine-functionalized clays							
Specific surface area (m^2^/g)	41.3	29.0	4.0	0.7	26.8	50	30.0
Average pore radius (nm)	25.20	12.08	13.52	3.78	11.27	5.96	10.79

The specific surface areas were calculated by the BET equation (*S*_BET_) and are listed in Table 4. Original clays exhibited higher surface areas compared to functionalized clays with the highest value of ca. 137 m^2^/g for clay A. On the other hand, clays C and D showed low surface areas ranging ca. 7.5–35.0 m^2^/g for original clays and ca. 0.7–4.0 m^2^/g for amine-functionalized clays. The expected decreased surface area on the modified clays confirmed the impregnation of the amines in the clay structures. For the functionalized clays, clay F showed the highest surface area of ca. 50 m^2^/g. In the case of original clays (industrial and artisanal usage), there was no clear correlation between the *S*_BET_ and the CO_2_ loading which might be ascribed to the different mineralogical composition for the seven samples.

#### 3.3.2 X-ray Diffraction (XRD)

X-ray diffraction was used to determine the composition, and crystalline phases present in the clay-based materials. Diffraction results were obtained for the samples before (original clays) and after the reaction with CO_2_, considering the samples with and without functionalization, labeled raw clays and amine-functionalized clays, respectively. This analysis was performed to determine if any changes in composition occurred in the different cases. Tables 5 and 6 show the clay samples’ relative abundance (%) for industrial and artisanal usage, respectively.

**Table 5:** Relative Abundance of Compounds in Different Industrial Usage Clays

		Relative Abundance (%)											
		Clay A			Clay B			Clay C			Clay D		
Compound	Formula	Original clay^a^	Raw clay	Amine-functionalized clay	Original clay^a^	Raw clay	Amine-functionalized clay	Original clay^a^	Raw clay	Amine-functionalized clay	Original clay^a^	Raw clay	Amine-functionalized clay
Calcite	CaCO_3_	0.34	0.08	0.29	0.17	8.14	0.41	0.25	0.47	0.28	-^b^	3.78	0.55
Dolomite	CaMg(CO_3_)_2_	1.29	1.33	1.31	0.58	0.51	0.72	0.19	1.20	0.88	0.96	0.65	1.06
Quartz	SiO_2_	0.21	0.22	0.23	2.48	1.38	1.64	1.00	1.35	1.70	3.15	3.22	3.10
Kaolinite	Al_2_(Si_2_O_5_)(OH)_4_	34.91	31.30	33.35	60.05	41.50	59.00	59.96	40.54	52.95	47.75	40.24	41.29
Albite	Na(AlSi_3_O_8_)	3.23	2.03	1.81	5.31	4.58	5.76	2.64	3.22	1.67	0.37	2.35	4.69
Anorthite	Ca(Al_2_Si_2_O_8_)	2.70	1.43	3.97	1.55	1.32	2.79	1.96	5.98	4.63	4.37	5.02	5.74
Hornblende	(Ca_2_[(Fe)_4_Al](Si_7_Al)O_22_(OH)_2_)	0.33	0.19	0.56	1.19	3.20	2.57	1.21	3.38	2.06	6.51	4.35	4.94
Hematite	Fe_2_O_3_	0.93	1.18	1.21	0.47	1.60	0.61	0.79	1.79	0.84	-^b^	1.87	1.83
Anatase	TiO_2_	0.08	-	0.09	0.98	0.09	0.95	0.05	-^b^	0.67	0.06	-^b^	0.07
Rutile	TiO_2_	0.28	0.20	0.43	0.95	0.41	0.28	0.65	0.68	0.72	0.65	0.06	0.65
Andesine	(Na,Ca)[Al(Si,Al)Si_2_O_8_]	50.28	56.36	50.78	24.88	33.94	23.91	28.47	38.23	32.62	32.35	34.19	31.66
Diopside	CaMgSi_2_O_6_	5.44	5.67	5.90	0.83	3.15	0.49	2.52	3.06	0.41	3.16	3.56	4.08
Makatite	Na_2_Si_4_O_8_(OH)_2_·4H_2_O	-^b^	-^b^	0.09	0.55	0.18	0.87	0.31	0.11	0.58	0.68	0.71	0.35

a Unreacted samples (before CO_2_ sorption).

b Not present.

**Table 6:** Relative Abundance of Compounds in Different Artisanal Usage Clays

		Relative Abundance (%)								
		Clay E			Clay F			Clay G		
Compound	Formula	Original clay^a^	Raw Clay	Amine-functionalized Clay	Original Clay^a^	Raw Clay	Amine-functionalized Clay	Original Clay^a^	Raw Clay	Amine-functionalized Clay
Calcite	CaCO_3_	-^b^	0.33	0.50	0.33	0.27	0.10	-^b^	-^b^	0.10
Dolomite	CaMg(CO_3_)_2_	-^b^	0.37	0.15	-^b^	0.67	0.33	-^b^	1.17	0.14
Quartz	SiO_2_	3.49	5.02	2.77	3.21	3.56	2.34	3.31	4.29	2.81
Kaolinite	Al_2_(Si_2_O_5_)(OH)_4_	85.35	68.98	83.02	62.78	53.17	63.60	65.41	49.64	67.09
Albite	Na(AlSi_3_O_8_)	1.17	5.19	3.79	5.27	4.25	6.08	1.61	5.97	4.11
Anorthite	Ca(Al_2_Si_2_O_8_)	0.43	3.61	-^b^	4.12	4.63	3.11	1.87	5.81	4.60
Hornblende	(Ca_2_[(Fe)_4_Al](Si_7_Al)O_22_(OH)_2_)	2.34	3.79	1.84	1.82	4.45	2.01	3.44	4.54	2.19
Hematite	Fe_2_O_3_	2.79	7.28	2.77	1.15	2.68	1.35	1.86	3.49	1.45
Anatase	TiO_2_	1.56	1.15	1.07	0.92	0.26	0.89	1.06	0.21	0.57
Rutile	TiO_2_	0.43	1.28	0.67	0.87	1.30	1.20	0.44	1.11	0.87
Andesine	(Na,Ca)[Al(Si,Al)Si_2_O_8_]	1.22	2.05	1.80	18.33	22.85	17.09	19.32	21.56	14.75
Diopside	CaMgSi_2_O_6_	1.12	0.87	1.61	0.48	1.16	1.46	1.20	1.68	0.55
Makatite	Na_2_Si_4_O_8_(OH)_2_·4H_2_O	0.11	0.09	-^b^	0.73	0.75	0.45	0.49	0.54	0.76

a Unreacted samples (before CO_2_ sorption).

b Not present.

Table 5 shows the relative abundance of compounds in different industrial usage clays. The predominant compounds were kaolinite and andesine. The first compound belongs to kaolinite-serpentine group and the other is characteristic of the feldspar group (plagioclase family). On the other hand, secondary phases such as quartz, hematite, hornblende, anatase, andesine, albite, diopside, rutile, anorthite, makatite, dolomite, and calcite were also observed.^93^ Although phyllosilicates are the most representative minerals in clays, it was evident that in these samples.^94–96^ In addition, the only major mineral phase that differed from this trend was in clay A, where andesine and kaolinite were the majority, and as crystalline phases secondary were diopside, albite, anorthite, dolomite and so on.^94–96^ In the case of the samples CO_2_-reacted with amine-functionalization, mineral phase identification was observed similar to that of nonfunctionalized clays, maintaining the distribution of crystalline phases. An important aspect observed in the reacted, functionalized, and functionalized-reacted clays was the change in the crystalline phase distribution. However, this is not relevant for the reaction that fixes CO_2_ in the form of stable minerals.^97,98^

In addition, Table 6 shows the relative abundance of compounds in different artisanal usage clays. The results of the crystalline phase distribution were similar to those of industrial samples, where kaolinite is the majority, along with andesine in the original clays, followed by an abundant presence of components such as hematite, quartz, and anatase.^99^ Changes in mineral distribution were observed in the samples reacted with and without functionalization. These changes might result from the functionalization process, wherein the acidic amine environment increases the porosity and exposes more active sites of the material. In this way, the chemical interaction between CO_2_ and the mineral surface could be improved.^99^

#### 3.3.3 FTIR Analysis

The FTIR analysis identified the main functional groups in the clay, confirming amine functionalization based on the study of chemical interactions between CO_2_ and amines and the formation of carbamates. Figure 7 shows the results for clay A (industrial usage) and clay G (artisanal usage). It can be noted that the results of this analysis were similar to rest of clays (see Figure S5 for unreacted amine-functionalized clays).

**Figure 7:**
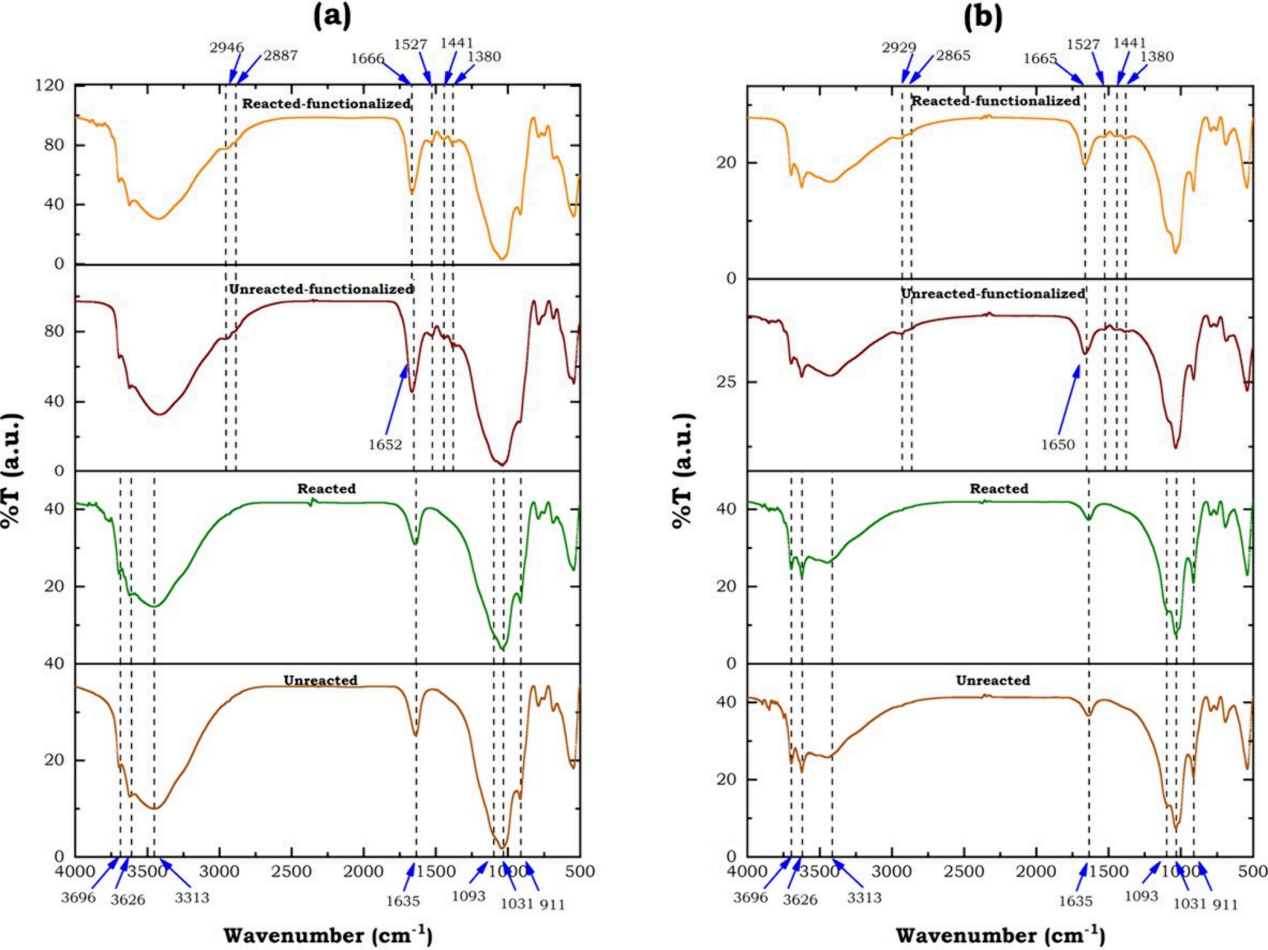
FTIR analysis results: (a) clay A (industrial usage); (b) clay G (artisanal usage clay).

Each sample—unreacted clay, clay CO_2_-reacted without functionalization, and clay CO_2_-reacted with functionalization—displayed spectral characteristics that reflect their molecular interactions and modifications. In their natural state, the unreacted clays exhibited fundamental absorption bands that define their crystalline matrix. The broad, prominent peaks between 3600–3700 cm^–1^ arose from the stretching vibrations of structural hydroxyl (−OH) of silanol groups embedded within the clay’s layered sheets. Meanwhile, the 1000–1100 cm^–1^ region showcased the strong, defining signatures of Si–O–Si bonds, indicative of the silicate framework. Additionally, the 800–900 cm^–1^ range revealed contributions from Al–OH and Al–O–Si linkages, further confirming the clay’s inherent mineral composition. The CO_2_-reacted clays without functionalization retained their spectral profile and remained largely unchanged, retaining their principal bands as the unreacted clays. However, subtle variations emerged most notably, a slight shift or diminished intensity in the 3600–3700 cm^–1^ region, due to partial dehydration. Significantly, the absence of new transmission peaks confirmed that no additional functional groups were introduced, leaving the fundamental structure of clays without alterations concerning unreacted clays.

During the amine-functionalization of both industrial and artisanal clays, the −NH_2_ or −OH groups of MEA/EDA interacted with the – OH group present on the clay surfaces. However, Figure 7 illustrates the distinctions between unreacted and reacted amine-functionalized clays. As seen in Figure 7, the unreacted clay exhibited characteristic adsorption bands typical of kaolinite. So, different authors thoroughly described these features in their studies, noting that the absorption peaks at 3696, 3626, and 3313 cm^–1^ are attributed to the stretching of the internal hydroxyl group, while the bands between 1093 and 911 cm^–1^ correspond to the bonding vibration stretching of Si–O.^100,101^ Additionally, following the amine-functionalization process, the bending peaks of −NH_2_ and −CH_2_– groups were observed at 1566 and 1495 cm^–1^, and at 2947 and 2870 cm^–1^, respectively. Furthermore, the clay A infrared spectra showed in the 1620–1720 cm^–1^ range a signal which increased after CO_2_ sorption, likely corresponding to carbamate (NCOO^–^) formation, a direct consequence of CO_2_ interaction with the functionalized clay. Although, this peak appeared around to 1660 cm^–1^, it resulted very weak but supported this hinting at the presence of carbonate species derived from CO_2_ adsorption. Finally, although the FTIR spectra of these clays may appear superficially similar, the delicate differences, particularly in the functionalized sample, provided compelling evidence of successful amine grafting and subsequent CO_2_ capture. The emergence of carbamate-related bands (1500–1700 cm^–1^) underscored the material’s potential for carbon sequestration applications, aligning with established findings in the literature.^102–104^

#### 3.3.4 TGA Analysis

The TGA analysis was used to evaluate the material’s thermal stability, analyze weight loss due to decomposition, and confirm the functionalization process with carbonate formation analysis. The analysis for clay G (artisanal usage clay) is shown in Figure 8. The analysis revealed distinct thermal degradation patterns across the samples. The unreacted clay experienced a total mass reduction of 12.2%. This reduction was characterized by gradual losses primarily occurring in two key temperature ranges: 4.7% between 150–400 °C and 5.3% between 400–600 °C. This pattern is characteristic of natural clays, where mass loss is due to the dehydration of interlayer water, followed by structural dehydroxylation.^35,87^ In contrast, the CO_2_-reacted clay exhibited the smallest total mass loss at 5.5%. This suggested that CO_2_ sorption enhances the thermal stability of the clay structure, as the marked decrease in all decomposition phases implies that the physically adsorbed CO_2_ within the interlayer space shielded the structure during heating. The amine-functionalized clay without CO_2_ showed the greatest total mass loss at 21.0%, with a particularly significant loss of 15.3% in the 150–400 °C range. This substantial loss could be attributed to the breakdown of the MEA and EDA amino functional groups attached to the clay surface. Finally, the amine-functionalized clay with CO_2_ sorption exhibited a similar total mass loss to that of sample at 20.8%, but it displayed slight variations in its decomposition profile, indicating interactions between the amino groups and the adsorbed CO_2_.^105,106^ The results of this work were similar for all clay samples (see Figure S6 and Figure S7).

**Figure 8:**
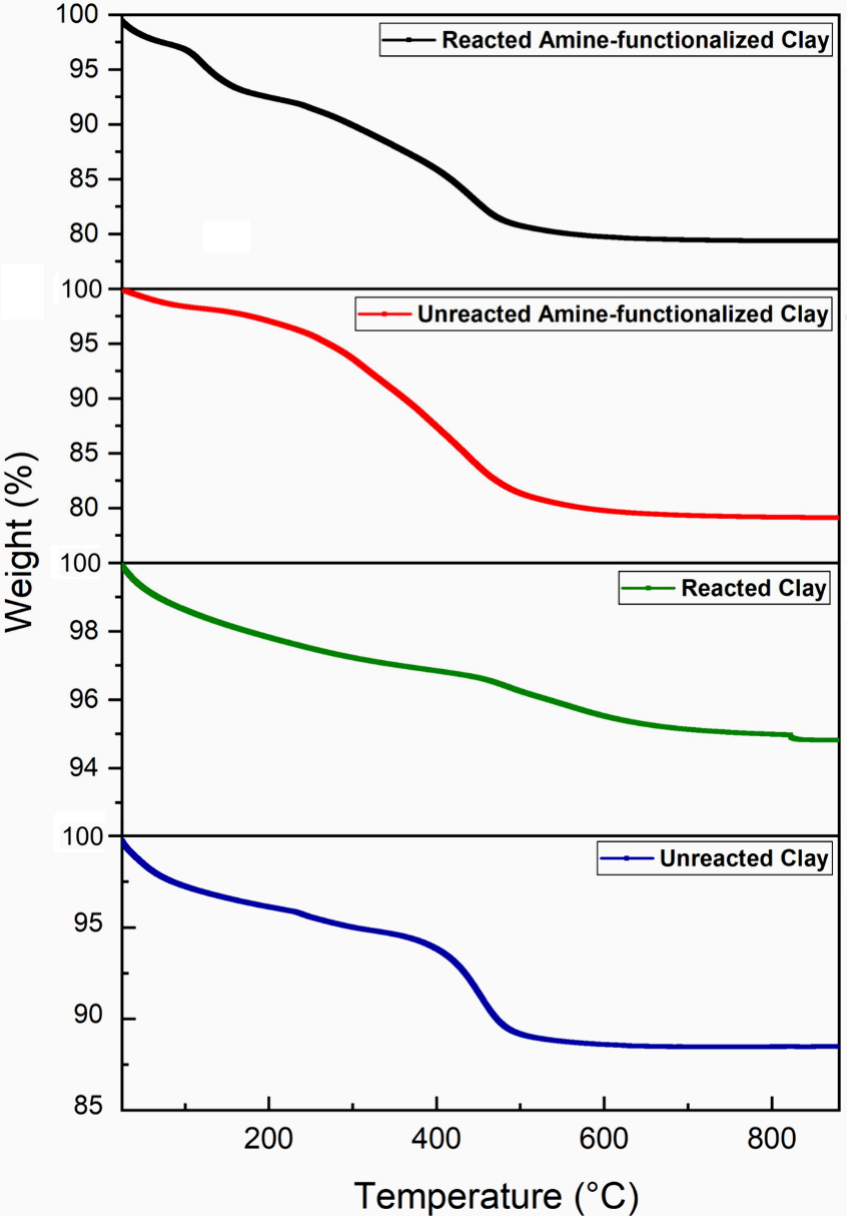
TGA analysis results: Clay G (artisanal usage clay).

#### 3.3.5 X-ray Fluorescence (XRF)

The XRF results obtained for the unreacted, reacted, unreacted and reacted amine-functionalized clay G with respect to the CO_2_ sorption capacity are highlighted in Table 7, which lists the percentage relative abundance of the chemical compositions of crystalline phases in terms of metal oxide quantities only for the crystalline phases majorities and for some minorities.^41,107^

**Table 7:** Relative Abundance of Compounds: Clay G (Artisanal Usage Clay)

	Relative Abundance (%)		
Metal Oxides	Original clay	Raw clay	Amine-functionalized clay
SiO_2_	53.63	54.36	53.57
Al_2_O_3_	29.48	28.68	28.76
Fe_2_O_3_	10.80	10.58	11.36
CaO	2.042	1.990	2.230
MgO	0.749	0.88	0.747
Na_2_O	1.120	1.316	1.077
K_2_O	0.532	0.551	0.530

The analysis of the major oxides revealed a remarkable stability in the structural matrix of the clay, where the concentrations of silica (SiO_2_) and alumina (Al_2_O_3_) did not present significant variations compared to the different treatments applied, such as 53.63% SiO_2_/29.48% Al_2_O_3_ for unreacted clay G, being similar to reacted and reacted amine-functionalized clays for sample G. This behavior suggested that the kaolinite and andesine, as the crystalline phase majorities of the sample clay, not suffer a significative change; therefore, this material functioned as a stable chemical and structural support. In stark contrast, alkaline oxides, specifically Na_2_O and K_2_O, exhibited distinct dynamics. The reacted clay induced the mobilization and slight concentration of these elements. However, the subsequent stage of functionalization reversed this phenomenon, resulting in a notable decrease in their concentrations, until reaching a lower value than the starting material in the case of Na_2_O. This behavior suggested that the functionalization mechanism could involve an ion exchange process.^108^

The XRF results provided direct evidence of changes in the chemical composition of the sample clays after the CO_2_ sorption reaction versus the same reaction with amine-functionalized clays. Another change registered by the XRF analysis was the dynamics of the CaCO_3_ (calcite) content across the sample set, knowing that the percentage relative abundance is directly proportional to CaO. However, it was not dependent only on that because, according to XRD analysis, the CaCO_3_ levels were not notably high—for instance, unreacted clay A (6.155%) and clay B (2.04%) have a major content of CaO, although these quantities were not only dependent on calcite but on all crystalline phases that contain calcium in their chemical composition (see Table S3 and Table S4).

## 4 Conclusion and Final Remarks

This work evaluated different clay-based materials from the Imbabura province (Ecuador) to identify good candidates for CO_2_ sorption. Different factors were considered, such as physicochemical properties, amine functionalization, and characterization techniques. The findings revealed that both industrial and artisanal clays were effective for CO_2_ capturing. The best results were obtained from the top layers of the industrial usage clays C and D, which achieved capture values of 0.980 mmol CO_2_/g clay and 0.933 mmol CO_2_/g clay, respectively. The best samples of raw clay for artisanal usage E and G managed to capture 1.008 mmol CO_2_/g clay and 0.634 mmol CO_2_/g clay, respectively. These results demonstrated that these materials were competitive with those reported in the literature.

A functionalization process was applied to enhance the CO_2_ sorption capacity, based on the wet impregnation method with monoethanolamine and ethylenediamine amines. The clay-rich portion (top layers) of the industrial usage clays were functionalized, which increased CO_2_ loading values, obtaining the best results in samples A and D with 1.396 mmol CO_2_/g clay and 1.547 mmol CO_2_/g clay, respectively. In contrast, better results were achieved for the artisanal usage clays F and G, with 2.663 mmol CO_2_/g clay and 3.125 mmol CO_2_/g clay, respectively. Thus, it can be assumed that the amine functionalization process was effective in improving the CO_2_ sorption capacity of these materials.

Several characterization techniques were used to analyze clay-based samples. Nitrogen physisorption isotherms showed the presence of mesoporosity on the clays and the comparison of specific surface areas between raw and amino-functionalized clays proves the impregnation of amines in the clays’ structure. With XRD, it was possible to determine the clays’ main mineral components before and after amine functionalization and to evaluate whether there was an expansion or intercalation of the amines in the lamellar structure. FTIR confirmed the incorporation of amines by detecting specific bands associated with amino groups, such as the vibrations of N–H and C–N bonds. Additionally, this technique verified the presence of the characteristic functional groups of the clay, such as the hydroxyl groups (−OH) associated with the phyllosilicates, which helps to evaluate the degree of chemical modification. Comparing the thermal curves of functionalized and nonfunctionalized clay from TGA analysis, it was possible to quantify the amount of organic material incorporated. Although changes in the ratio of metal oxides result in very similar small variations between pure clays and unreacted and reacted amino-functionalized clays. Even so, these small changes reflect an indication in the functionalization of clay and its subsequent reaction with CO_2_, showing the potential of these sorbents for CO_2_ capture.

The promising results obtained from this work indicate a viable path for using readily available, easy-to-process, and low-cost clays for CO_2_ capture and impact mitigation, thereby justifying extensive future research. Future research should focus on optimizing the process by exploring other conventional functionalization methods using various amines, while also considering the materials’ physicochemical properties, economic costs, and efficiency under industrial conditions. Key areas for continued study will be evaluating the regeneration of clays across multiple sorption–desorption cycles and conducting a detailed investigation into the effect of initial pressure and temperature on the CO_2_ capture process to optimize the overall system’s performance, particularly under a continuous capture setup.

## Supplementary Material



## Notes

Sampling sites, physical properties of the clays (density and plasticity), details on amine functionalization, CO_2_ sorption, and characterization data (FTIR, TGA, XRF) (PDF)
