# Supplementary material for: Amine-Functionalized Clays as Solid Sorbents: High-Pressure CO2 Sorption Testing and Characterization
Source: ACS Omega. 2026 Jan 13;11(3):3902–15. doi: 10.1021/acsomega.5c06923 (PMC12854600; doi:10.1021/acsomega.5c06923)
Supplement: Supplementary file 1 [file ao5c06923_si_001.pdf]

# Amine-Functionalized Clays as Solid Sorbents: High-Pressure $CO_2$ Sorption Testing and Characterization

Jennifer Narváez [1], Ernesto Bastardo-González [1], Edward E. Ávila [1], Alex Palma-Cando [1], Pamela Galarraga [2], Victor H. Guerrero [3], Marvin Ricaurte [1,*]

[1] Grupo de Investigación Aplicada en Materiales y Procesos (GIAMP), School of Chemical Sciences and Engineering, Yachay Tech University, Hacienda San José s/n y Proyecto Yachay, Urcuquí 100119, Ecuador.

[2] Research Development and Innovation Department, UNACEM Ecuador, Sector Perugachi Km 71 ½ vía a Selva Alegre, Otavalo, 110100, Ecuador.

[3] Department of Materials, Escuela Politécnica Nacional, Ladrón de Guevara E11-253, Quito 170525, Ecuador.

* Corresponding Author: mricaurte@yachaytech.edu.ec

# Supporting Information

## Location of Clay Samples

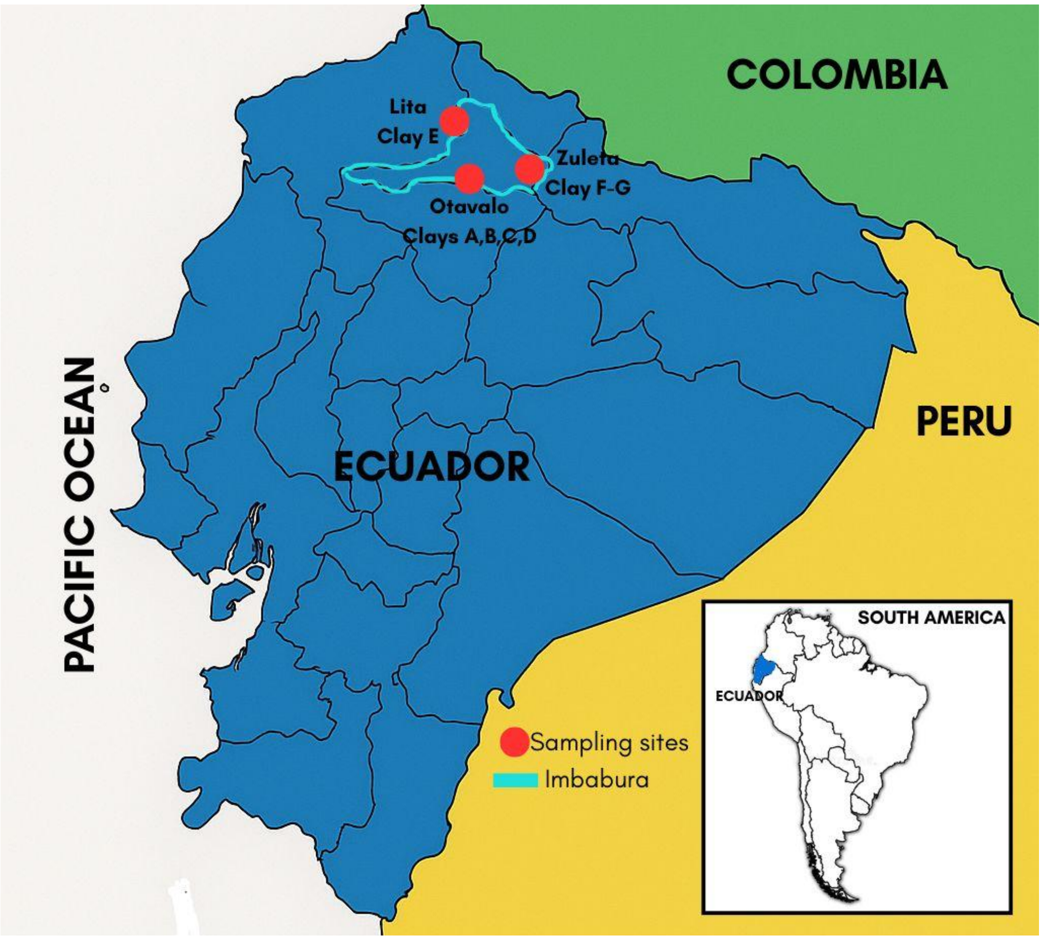


**Figure S1.** Location of clay-based materials sampling sites on the map of Ecuador.

# Density of the Clay-Based Materials

**Table S1.** Density of the clay-based materials.

| Samples | | Density (g/mL) |
|---|---|---|
| **Clay A** | Raw clay | 1.210 |
| | Top layer | 1.017 |
| | Middle layer | 1.005 |
| | Bottom layer | 1.315 |
| | Amine-functionalized clay | 0.919 |
| **Clay B** | Raw clay | 0.980 |
| | Top layer | 0.688 |
| | Middle layer | 0.825 |
| | Bottom layer | 0.989 |
| | Amine-functionalized clay | 0.836 |
| **Clay C** | Raw clay | 1.386 |
| | Top layer | 0.768 |
| | Middle layer | 0.834 |
| | Bottom layer | 1.167 |
| | Amine-functionalized clay | 0.819 |
| **Clay D** | Raw clay | 1.473 |
| | Top layer | 0.862 |
| | Bottom layer | 1.502 |
| | Amine-functionalized clay | 1.042 |
| **Clay E** | Raw clay | 0.778 |
| | Amine-functionalized clay | 0.842 |
| **Clay F** | Raw clay | 1.132 |
| | Amine-functionalized clay | 0.932 |
| **Clay G** | Raw clay | 0.980 |
| | Amine-functionalized clay | 1.014 |

## Plasticity of Different Layers of Industrial Usage Clays

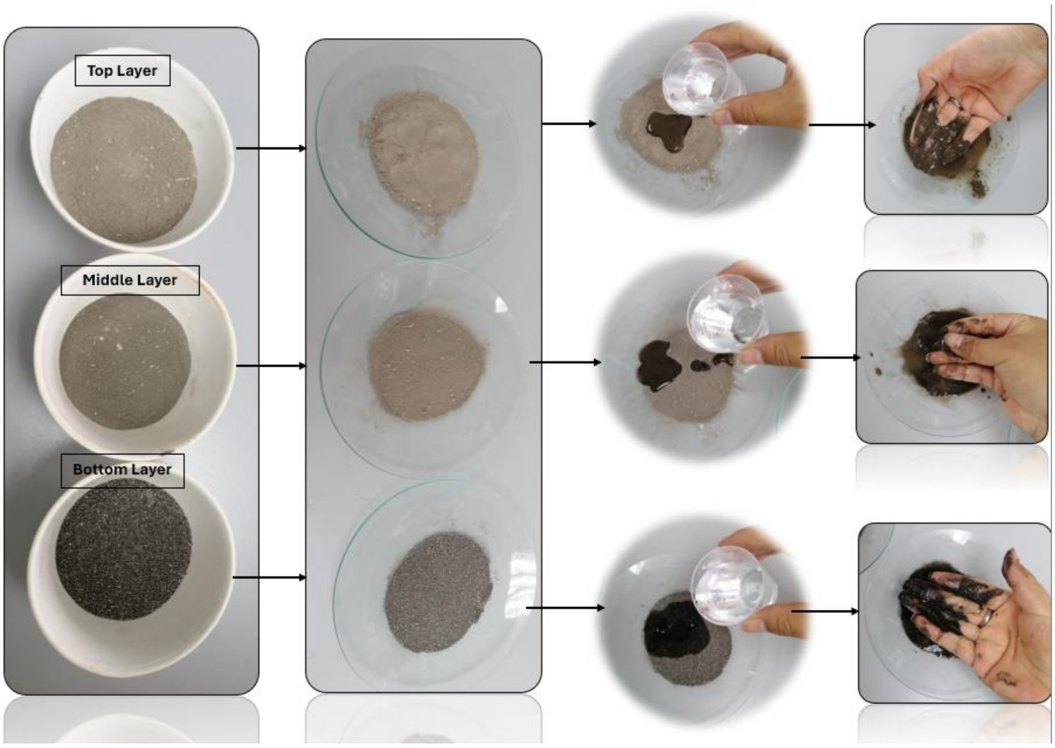


**Figure S2.** The plasticity effect of different layers of industrial usage clays.

## Amine-Functionalization Process: Wet Impregnation Method

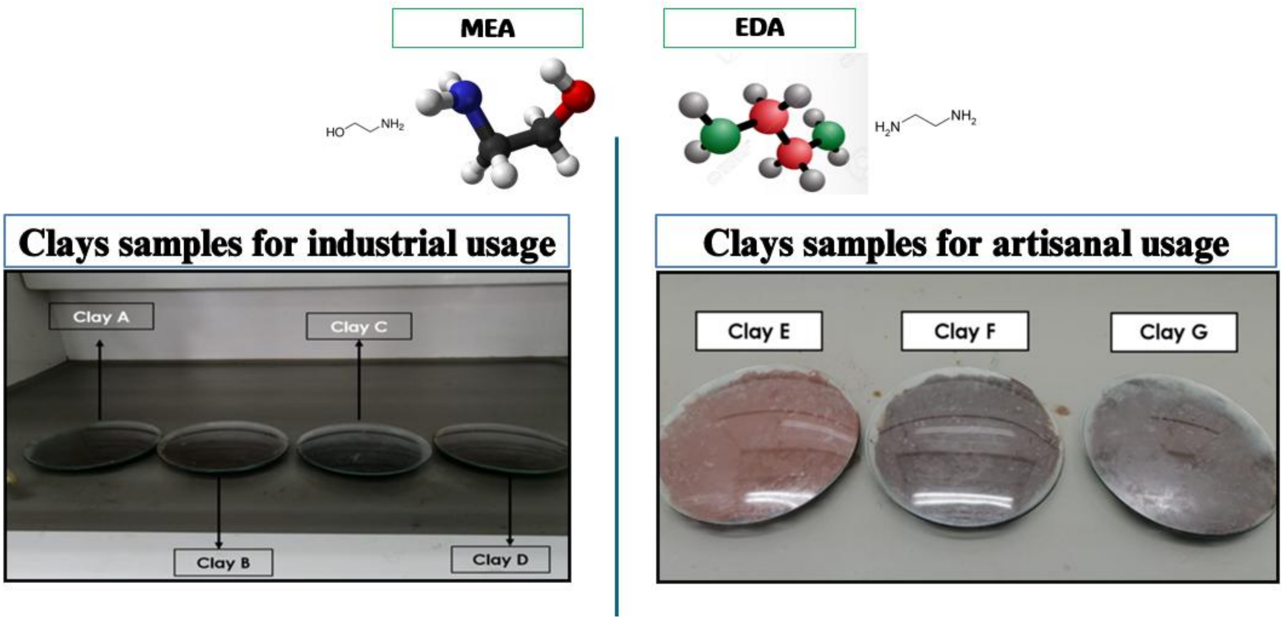


**Figure S3.** Amine-functionalized clays: wet impregnation method.

**Table S2.** Amine-functionalized clays: mass increase due to wet impregnation method.

| Samples | Amine content (g-amine / g clay) | Mass Increase (%) |
|---|---|---|
| **Clay A** | 0.067 | 6.68 |
| **Clay B** | 0.080 | 8.03 |
| **Clay C** | 0.033 | 3.30 |
| **Clay D** | 0.075 | 7.51 |
| **Clay E** | 0.010 | 1.02 |
| **Clay F** | 0.059 | 5.90 |
| **Clay G** | 0.066 | 6.56 |

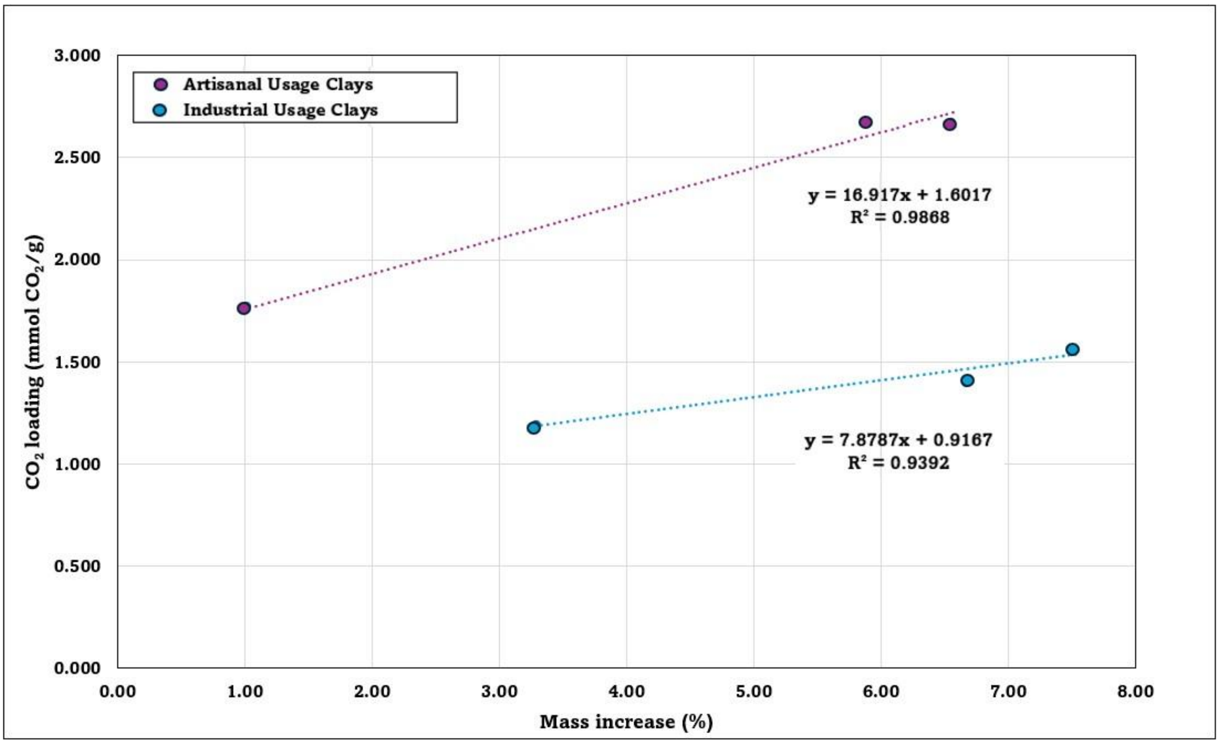


**Figure S4.** $CO_2$ loading vs. mass increase (amine-functionalized clays).

## FTIR Analysis

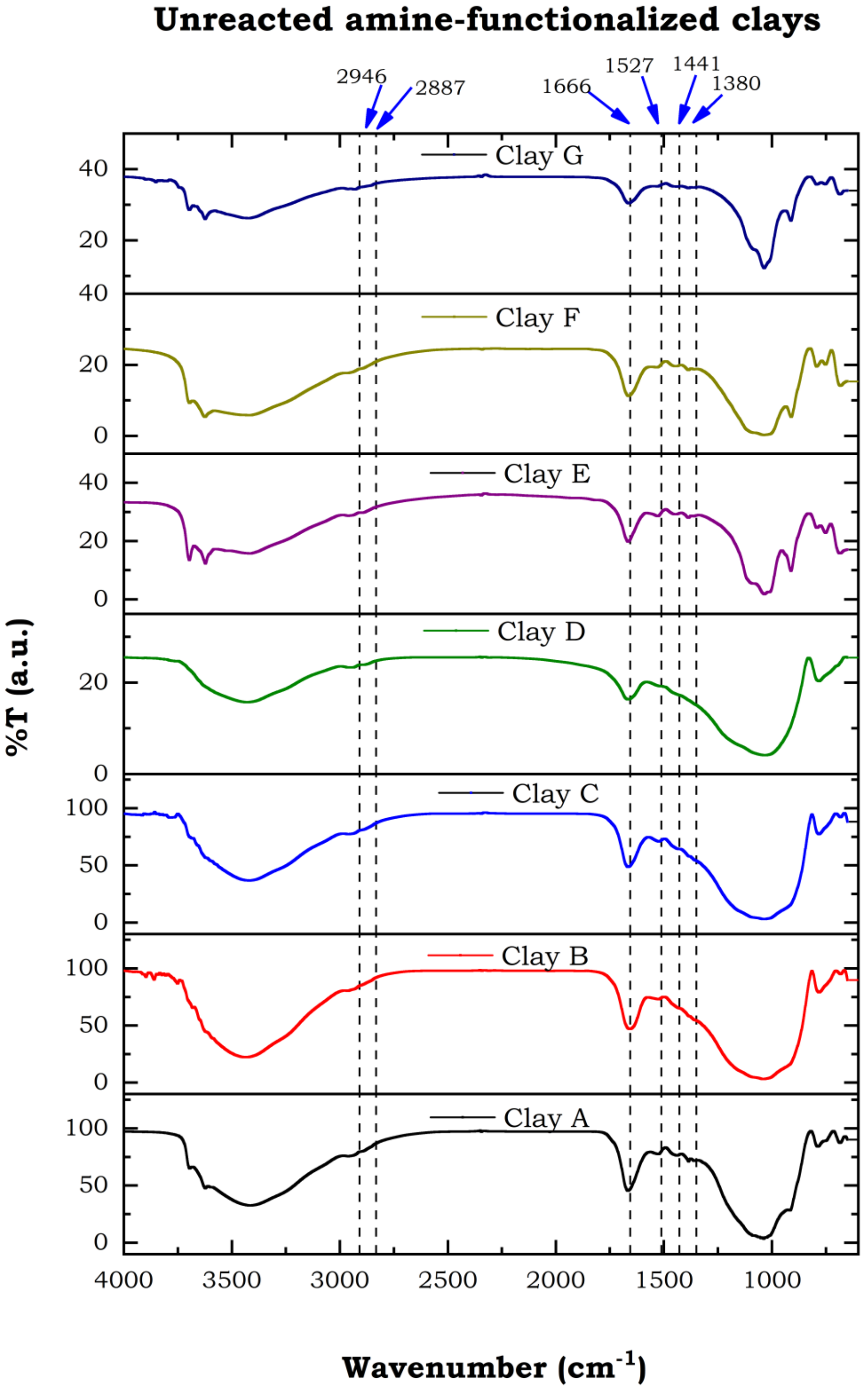


**Figure S5.** FTIR spectra of unreacted amine-functionalized clays. Only the bands corresponding to the stretching vibrations of the amine supported the different clays.

# TGA Analysis

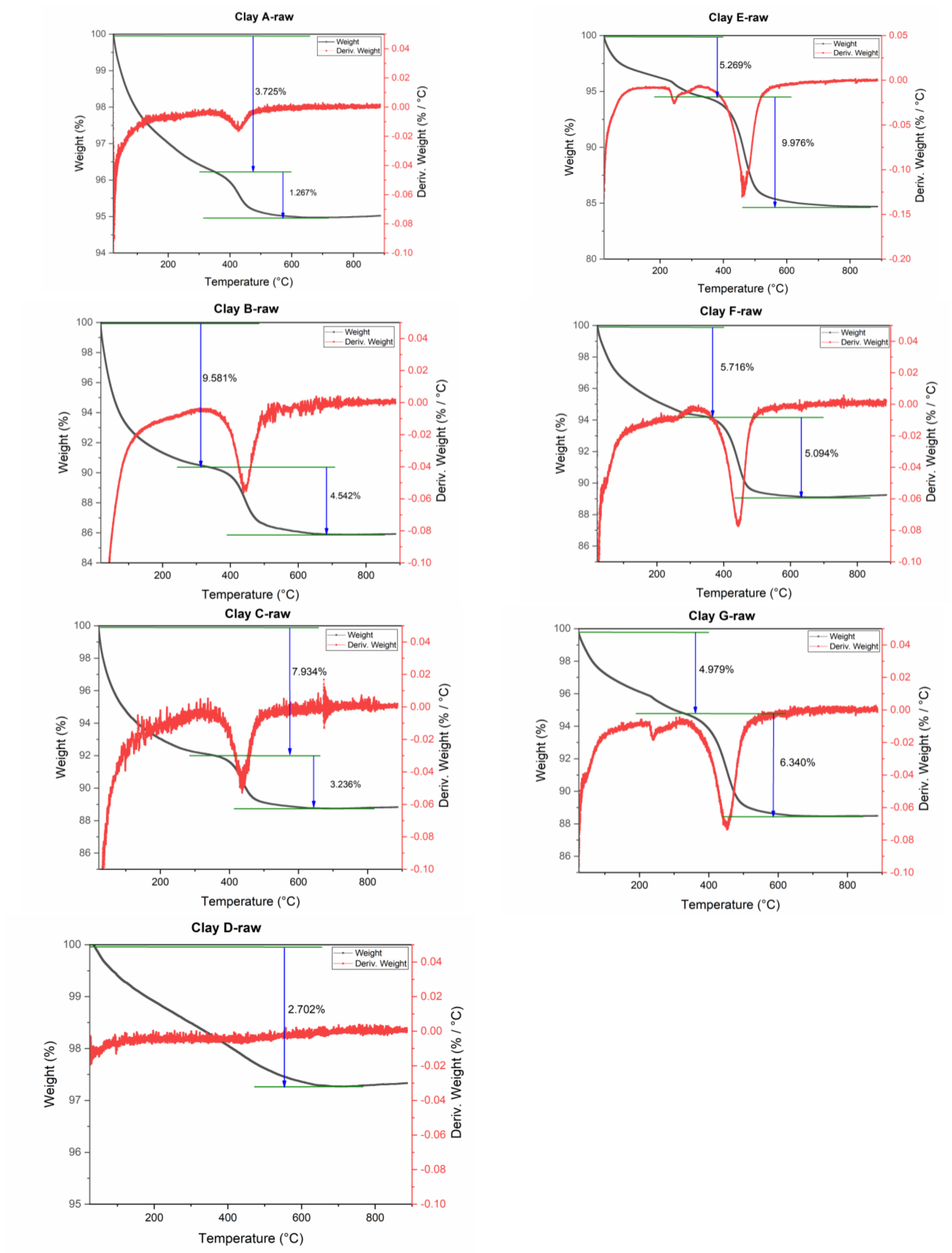


**Figure S6.** TGA plots for unreated clays.

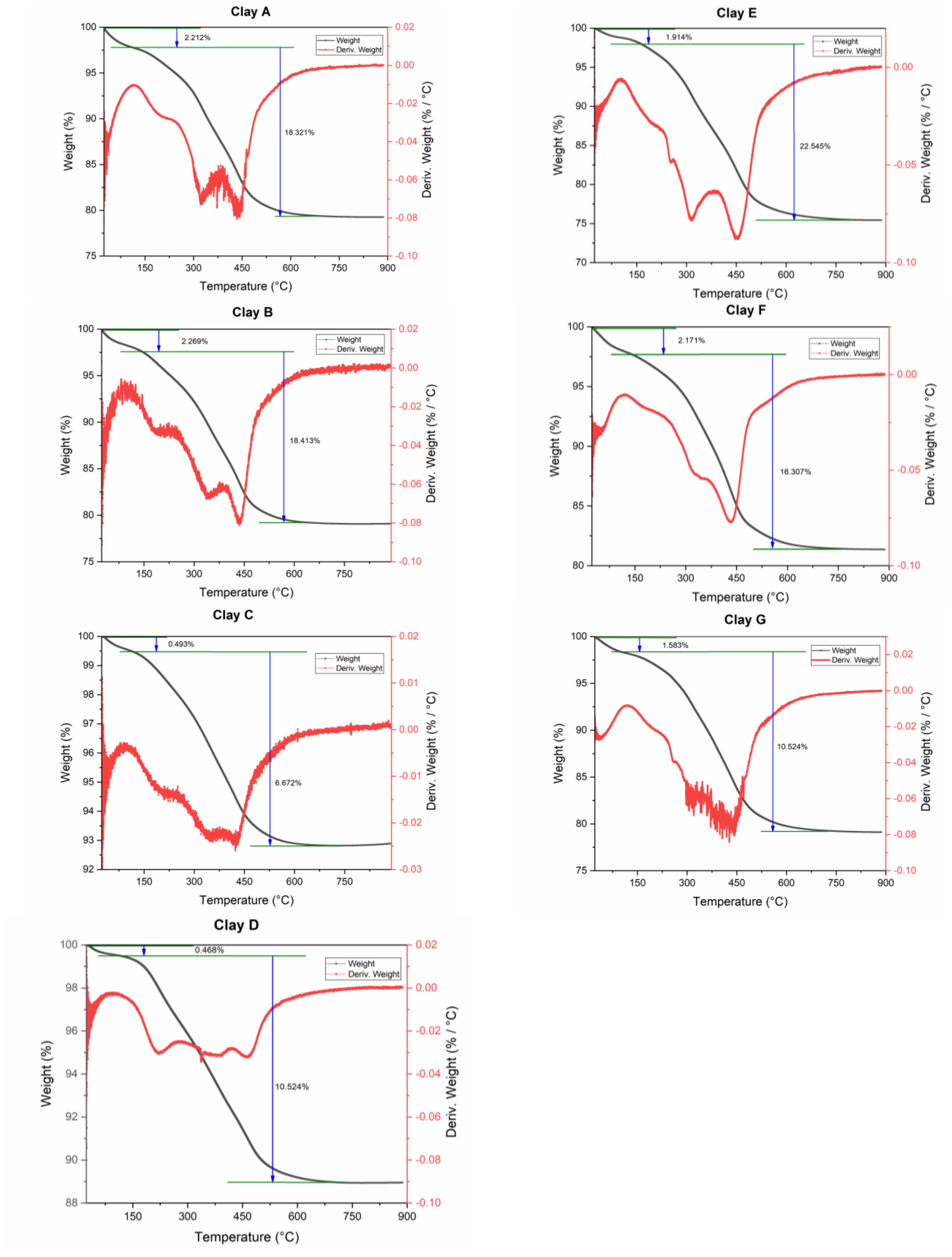


**Figure S7.** TGA plots for amine-functionalized clays.

## XRF Analysis

**Table S3.** Percentage relative abundance (%) of chemical composition in metal oxides for compounds in different industrial usage clays.

| | RELATIVE ABUNDANCE (%) | | | | | | | | | | | |
|---|---|---|---|---|---|---|---|---|---|---|---|---|
| | Clay A | | | Clay B | | | Clay C | | | Clay D | | |
| Compound | Unreacted | Reacted | Reacted Amine-functionalized | Unreacted | Reacted | Reacted Amine-functionalized | Unreacted | Reacted | Reacted Amine-functionalized | Unreacted | Reacted | Reacted Amine-functionalized |
| $SiO_2$ | 59.680 | 59.130 | 59.580 | 59.570 | 59.470 | 59.250 | 61.070 | 61.810 | 60.350 | 65.028 | 65.788 | 65.330 |
| $Al_2O_3$ | 19.390 | 19.610 | 19.120 | 25.170 | 25.430 | 24.840 | 21.370 | 22.550 | 21.540 | 16.220 | 16.290 | 16.340 |
| $Fe_2O_3$ | 6.457 | 6.490 | 6.350 | 7.640 | 7.600 | 7.840 | 7.920 | 7.105 | 7.640 | 5.570 | 4.690 | 4.870 |
| CaO | 6.155 | 6.240 | 6.390 | 2.830 | 2.730 | 3.220 | 4.440 | 3.267 | 4.970 | 4.880 | 4.530 | 4.940 |
| MgO | 1.960 | 2.037 | 2.091 | 1.677 | 0.807 | 0.782 | 0.877 | 0.942 | 0.952 | 1.790 | 1.930 | 1.866 |
| $SO_3$ | 0.117 | 0.076 | 0.064 | 0.062 | 0.043 | 0.041 | 0.099 | 0.041 | 0.097 | 0.043 | - | 0.035 |
| $Na_2O$ | 3.676 | 3.961 | 3.910 | 1.677 | 1.757 | 1.737 | 1.822 | 2.044 | 1.990 | 3.528 | 4.053 | 3.900 |
| $K_2O$ | 1.338 | 1.299 | 1.340 | 0.900 | 0.846 | 0.966 | 1.038 | 1.026 | 1.096 | 1.877 | 1.825 | 1.807 |
| $TiO_2$ | 0.666 | 0.656 | 0.663 | 0.782 | 0.775 | 0.795 | 0.745 | 0.712 | 0.751 | 1.791 | 0.393 | 0.400 |
| $P_2O_5$ | 0.177 | 0.152 | 0.140 | 0.186 | 0.189 | 0.175 | 0.192 | 0.169 | 0.174 | 0.175 | 0.130 | 0.154 |
| BaO | 0.093 | 0.084 | 0.086 | 0.158 | 0.125 | 0.142 | 0.151 | 0.138 | 0.151 | 0.125 | 0.091 | 0.075 |
| MnO | 0.079 | 0.085 | 0.077 | 0.080 | 0.078 | 0.085 | 0.090 | 0.085 | 0.090 | 0.103 | 0.093 | 0.101 |
| SrO | 0.068 | 0.063 | 0.066 | 0.039 | 0.036 | 0.041 | 0.045 | 0.040 | 0.045 | 0.055 | 0.049 | 0.051 |
| $ZrO_2$ | 0.034 | 0.026 | 0.026 | 0.021 | 0.020 | 0.022 | 0.041 | 0.014 | 0.037 | 0.030 | 0.032 | 0.025 |
| $Cr_2O_3$ | 0.027 | 0.030 | 0.028 | 0.021 | 0.021 | 0.011 | 0.023 | 0.017 | 0.017 | 0.046 | 0.019 | 0.020 |
| Cl | 0.027 | 0.016 | 0.019 | - | 0.014 | - | 0.025 | - | 0.024 | 0.035 | 0.039 | 0.036 |
| CuO | 0.018 | 0.014 | 0.014 | 0.013 | 0.012 | 0.013 | 0.014 | 0.013 | 0.013 | 0.017 | 0.014 | 0.014 |
| ZnO | 0.018 | 0.011 | 0.011 | 0.014 | 0.013 | 0.016 | 0.016 | 0.013 | 0.015 | 0.013 | 0.011 | 0.010 |
| NiO | 0.020 | 0.005 | 0.005 | 0.006 | 0.007 | 0.000 | 0.007 | 0.007 | 0.008 | 0.006 | 0.005 | 0.004 |

(–) Not present.

**Table S4.** Percentage relative abundance (%) of chemical composition in metal oxides for compounds in different artisanal usage clays.

| RELATIVE ABUNDANCE (%) | | | | | | | | | |
|---|---|---|---|---|---|---|---|---|---|
| | Clay E | | | Clay F | | | Clay G | | |
| Compound | Unreacted | Reacted | Reacted Amine-functionalized | Unreacted | Reacted | Reacted Amine-functionalized | Unreacted | Reacted | Reacted Amine-functionalized |
| $SiO_2$ | 47.230 | 47.880 | 47.880 | 59.017 | 57.540 | 54.546 | 53.630 | 54.360 | 53.570 |
| $Al_2O_3$ | 35.230 | 34.090 | 34.110 | 24.769 | 25.450 | 27.621 | 29.480 | 28.680 | 28.760 |
| $Fe_2O_3$ | 14.800 | 14.920 | 14.860 | 9.800 | 10.050 | 10.955 | 10.800 | 10.580 | 11.360 |
| CaO | 0.411 | 0.482 | 0.621 | 2.360 | 2.320 | 2.379 | 2.042 | 1.990 | 2.230 |
| MgO | 0.173 | 0.227 | 0.208 | 0.829 | 1.028 | 0.930 | 0.749 | 0.880 | 0.747 |
| $SO_3$ | 0.190 | 0.172 | 0.169 | 0.063 | 0.072 | 0.065 | 0.123 | 0.098 | 0.145 |
| $Na_2O$ | 0.147 | 0.348 | 0.240 | 1.068 | 1.360 | 1.168 | 1.120 | 1.316 | 1.077 |
| $K_2O$ | 0.092 | 0.113 | 0.117 | 0.584 | 0.660 | 0.669 | 0.532 | 0.551 | 0.530 |
| $TiO_2$ | 1.381 | 1.420 | 1.447 | 1.033 | 1.034 | 1.127 | 1.117 | 1.099 | 1.142 |
| $P_2O_5$ | 0.081 | 0.089 | 0.084 | 0.066 | 0.087 | 0.080 | 0.097 | 0.092 | 0.083 |
| BaO | 0.044 | 0.039 | 0.044 | 0.109 | 0.099 | 0.131 | 0.058 | 0.091 | 0.084 |
| MnO | 0.041 | 0.045 | 0.047 | 0.112 | 0.111 | 0.122 | 0.077 | 0.080 | 0.080 |
| SrO | 0.002 | 0.003 | 0.003 | 0.034 | 0.035 | 0.040 | 0.030 | 0.029 | 0.031 |
| $ZrO_2$ | 0.034 | 0.034 | 0.032 | 0.029 | 0.031 | 0.034 | 0.026 | 0.027 | 0.027 |
| $Cr_2O_3$ | 0.012 | 0.020 | 0.015 | 0.020 | 0.025 | 0.016 | 0.016 | 0.012 | 0.019 |
| Cl | 0.017 | 0.000 | 0.018 | 0.015 | 0.000 | 0.018 | 0.014 | 0.018 | 0.017 |
| CuO | 0.012 | 0.015 | 0.014 | 0.011 | 0.013 | 0.012 | 0.011 | 0.010 | 0.011 |
| ZnO | 0.013 | 0.015 | 0.014 | 0.014 | 0.014 | 0.018 | 0.011 | 0.012 | 0.013 |
| NiO | 0.010 | 0.010 | 0.010 | 0.007 | 0.007 | 0.009 | 0.008 | 0.008 | 0.007 |

(–) Not present.
